# Prevention of Early Postoperative Decline (PEaPoD): protocol for a randomized, controlled feasibility trial

**DOI:** 10.1186/s13063-018-3063-z

**Published:** 2018-12-11

**Authors:** Brian O’Gara, Edward R. Marcantonio, Alvaro Pascual-Leone, Shahzad Shaefi, Ariel Mueller, Valerie Banner-Goodspeed, Daniel Talmor, Balachundhar Subramaniam

**Affiliations:** 1 Department of Anesthesia, Critical Care and Pain Medicine, Beth Israel Deaconess Medical Center, Harvard Medical School, 330 Brookline Ave, Boston, MA 02215 USA; 2 Division of General Medicine and Primary Care, Beth Israel Deaconess Medical Center, Harvard Medical School, 330 Brookline Ave, Boston, MA 02215 USA; 3 Division of Neurology, Beth Israel Deaconess Medical Center, Harvard Medical School, 330 Brookline Ave, Boston, MA 02215 USA

**Keywords:** Delirium, Postoperative cognitive decline, Cardiac surgery, Neurocognitive training, Confusion Assessment Method, Montreal Cognitive Assessment

## Abstract

**Background:**

Delirium is associated with a significantly increased risk of postoperative morbidity and mortality. Furthermore, delirium has been associated with an increased risk of prolonged cognitive deficits and accelerated long-term cognitive decline. To date, experimental interventions for delirium have mainly focused on alternative pharmacologic and behavioral strategies in the postoperative period. Few studies have examined whether proactive strategies started *before* surgery can prevent delirium or reduce its sequelae. Neurocognitive training programs such as Lumosity have been shown to be effective in increasing cognitive performance in both elderly healthy volunteers and patients suffering from a myriad of acute and chronic medical conditions. When initiated in the preoperative period, such training programs may serve as interesting and novel patient-led interventions for the prevention of delirium and postoperative cognitive decline (POCD). We hypothesize that perioperative neurocognitive training is feasible in the older cardiac surgical population and are testing this hypothesis using a randomized controlled design.

**Methods:**

The Prevention of Early Postoperative Decline (PEaPoD) study is a randomized, controlled trial with a target enrollment of 45 elderly cardiac surgical patients. Subjects will be randomized in a 1:1 ratio to undergo either at least 10 days of preoperative neurocognitive training, continued for 4 weeks postoperatively, or usual care control. The primary outcome, feasibility, will be assessed by study recruitment and adherence to protocol. Secondary outcomes will include potential differences in the incidence of postoperative in-hospital delirium and POCD up to 6 months, as determined by the Confusion Assessment Method and the Montreal Cognitive Assessment.

**Discussion:**

PEaPoD will be the first trial investigating the use of perioperative cognitive training to potentially reduce delirium and POCD in the cardiac surgical population. Information gleaned from this feasibility study will prove valuable in designing future efficacy studies aimed at determining whether this low-risk, patient-led intervention can reduce serious postoperative morbidity.

**Trial registration:**

ClinicalTrials.gov, NCT02908464. Registered on 21 September 2016.

**Electronic supplementary material:**

The online version of this article (10.1186/s13063-018-3063-z) contains supplementary material, which is available to authorized users.

## Background

Characterized by altered consciousness, disorientation, and inattention, delirium is associated with an increased incidence of postoperative complications, prolonged hospital length of stay, and greater in-hospital mortality [1]. The highest reported incidence of postoperative delirium is in those undergoing cardiac surgery, with nearly 200,000 patients per year in the USA experiencing this morbid condition during their hospital admission [2]. While classically defined as a temporary condition, recent data has demonstrated that many patients with delirium can experience long-lasting effects on their cognitive performance and an accelerated rate of long-term cognitive decline [1, 2]. Defined by persistent deficits in memory and executive function, postoperative cognitive decline (POCD) can be detected in roughly 30% of patients 6 months after the day of their operation [3, 4]. Given an aging population’s projected increasing demand for cardiac surgery and the significant impact of delirium and POCD on postoperative recovery, a simple, effective, low-risk intervention to reduce their incidence would be invaluable [5]. Numerous investigations have tested various strategies to prevent postoperative delirium and cognitive dysfunction, largely focusing on pharmacologic interventions, with varying degrees of success [6]. On the other hand, non-pharmacologic strategies such as the Hospital Elder Life Program (HELP) have shown consistent benefit in reducing the incidence of delirium and have gained widespread acceptance, particularly in medical patients [7]. Although HELP includes guidelines for a perioperative regimen that includes frequent cognitive stimulation, at this time there has not been a consensus as to the most effective modality.

In an effort to reproduce the gains observed with physical prehabilitation in improving postoperative functional recovery, clinicians and researchers have begun to consider cognitive prehabilitation’s potential to maximize recovery following major surgery [8, 9]. For example, preclinical data has demonstrated that preoperative cognitive enrichment can reduce perioperative neuronal inflammation and POCD in rats [10]. Additionally, data from observational studies in humans suggests that preoperative participation in cognitively stimulating activities is associated with a reduced incidence and severity of postoperative delirium [11]. Given these findings, it would follow that modalities that can potentially improve cognitive performance or build reserve in domains commonly affected by surgical exposure would serve as ideal candidate interventions for clinical trials evaluating their potential benefit in the older surgical population.

For adults over 60 years of age, normal aging is associated with declining cognitive ability, particularly in the executive functions of the prefrontal cortex, including attention, working memory, and processing speed [12]. Building on existing data suggesting improvement in these domains after video game use in young adults, there has been a push to develop interactive software aimed at similarly improving cognitive performance in older adults [13]. To date, multiple interactive platforms have emerged which package rigorous attention and memory tasks within engaging and entertaining gaming programs, and many have shown encouraging early results. For example, results of a secondary analysis of the data from the Advanced Cognitive Training for Independent and Vital Elderly (ACTIVE) trial suggest that the use of computerized brain training games can reduce the incidence and slow the onset of age-related dementia [14].

Lumosity, a commercially available neurocognitive gaming platform, has a promising early track record of improving cognitive performance in older adults. In a prospective randomized trial, training with Lumosity has been shown to reduce distraction and increase alertness, and it has been associated with significantly better performance on attention and both immediate and delayed visual memory tasks compared to controls [15, 16]. Additionally, when compared to an active control group, participants in another randomized controlled trial were found to have significantly greater improvements in speed of processing, short-term memory, working memory, problem solving, and fluid reasoning assessments [17]. Furthermore, as many of the same deficits found in age-related cognitive decline are present in both acute and chronic medical conditions, investigators have expanded the use of related neurocognitive training programs to rehabilitate cognitive dysfunction arising in numerous patient subgroups, including those with traumatic brain injury, stroke, and heart failure [18, 19]. Unlike these physical conditions, perioperative delirium and POCD occur with a known and predictable onset, making them amenable to preventive strategies. Therefore, the preoperative period provides an attractive target for a prehabilitative strategy to reduce the impact of these morbid conditions.

Despite promising preliminary data from allied fields, the concept of neurocognitive training in the perioperative period has not yet been investigated. The objective of this study is to determine the feasibility of using a perioperative neurocognitive training program in the cardiac surgical population. Data obtained through the conduct of this study will prove critical to the design and implementation of future protocols evaluating the potential efficacy of neurocognitive training to prevent postoperative delirium and POCD.

## Methods and design

### Study design

**Fig. 1:**
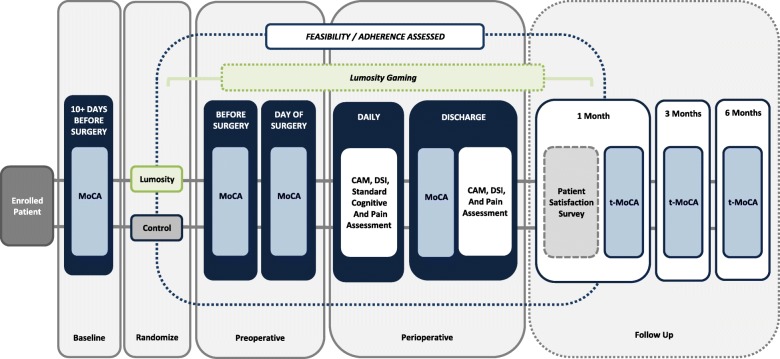
Study schema

**Fig. 2:**
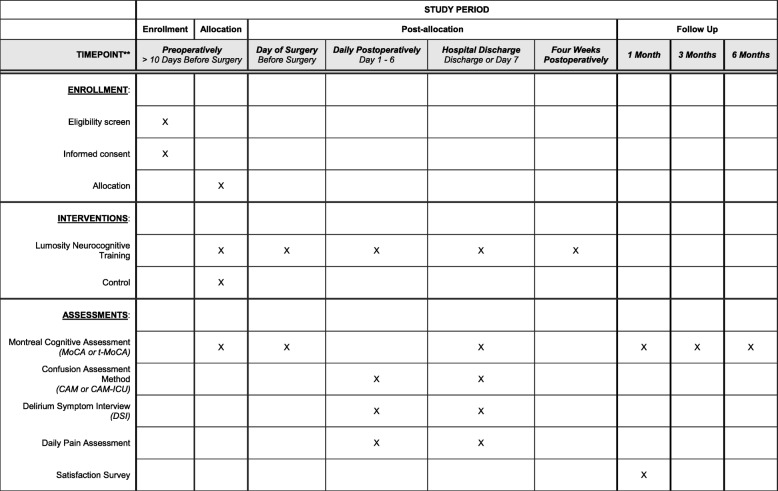
Standard Protocol Items: Recommendations for Interventional Trials reporting of study enrollment, interventions, and assessments

The Prevention of Early Postoperative Decline (PEaPoD) study is a randomized, controlled, single-center, assessor-blinded clinical trial of eligible older adult cardiac surgical patients. Subjects will be randomized in a 1:1 allocation to either perioperative neurocognitive training or usual care control. The primary outcome of feasibility will be determined based on enrollment and protocol adherence. Secondary outcomes, including the incidence of postoperative delirium and POCD, will be evaluated using the Confusion Assessment Method (CAM) and the Montreal Cognitive Assessment (MoCA) respectively. Additionally, patients will be followed until 6 months postoperatively and evaluated via a telephonic MoCA (t-MoCA) to assess the presence of long-term POCD and to map the trajectory of return to baseline cognitive function. A study schema is provided in Fig. 1. Our study protocol was written according to the principles outlined in the Standard Protocol Items: Recommendations for Interventional Trials (SPIRIT) checklist (Fig. 2, Additional file 1) [20].

### Setting

This study will be conducted at the Beth Israel Deaconess Medical Center (BIDMC) in Boston. BIDMC is a 700-bed tertiary care hospital academically affiliated with the Harvard Medical School. More than 900 open-heart procedures with cardiopulmonary bypass (CPB) are performed at BIDMC per year.

### Study registration

Institutional Review Board (IRB) approval was obtained, and oversight of the study by the BIDMC’s Committee on Clinical Investigations is currently ongoing under IRB #2016P000145. PEaPoD was registered with the identifier NCT02908464 on the National Institutes of Health (NIH) ClinicalTrials.gov website on 21 September 2016. Once the trial is completed, results will be reported according to the Consolidated Standards of Reporting Trials (CONSORT) guidelines and the SPIRIT checklist (Additional file 1) [20, 21]. The trial is active and ongoing, and any amendments made to the protocol are reported to and approved by the BIDMC IRB.

### Inclusion criteria

Patients will be deemed eligible for enrollment if they are between the ages of 60 and 90, are scheduled to undergo cardiac surgery at least 10 days after enrollment, and have obtained an educational level of at least high school or equivalent. The educational level requirement was chosen to help screen out pre-exisiting cognitive deficits and attempt to homogenize the baseline cogntive abilities of the study population. As neurocognitive training with Lumosity is thought to be most effective with greater training times, we chose a minimum of 10 days of lead-in time as an appropriate cutoff to balance the interests of an effective intervention with scheduling demands for cardiac surgery, which frequently dictate short preoperative wait times.

### Exclusion criteria

Patients will be excluded for a history of pre-existing psychiatric illness such as anxiety or depression, stroke, dementia, epilepsy, Parkinson’s disease, or other forms of cognitive decline. Due to the nature of the study intervention, non-English speaking patients and those with significant visual impairment will also be excluded.

### Dropout criteria

After obtaining consent, blinded study investigators will perform a baseline MoCA with the patient. Patients with a score of less than 10 (indicating severe baseline cognitive impairment) will be withdrawn from the study.

### Study intervention: neurocognitive training

Patients will be randomized in a 1:1 allocation via a concealed block scheme to either intervention or control using the Research Electronic Data Capture (REDCap) randomization feature. Patients randomized to the intervention group will be asked to complete two 15-min neurocognitive training sessions per day, from the day of enrollment until 4 weeks after their day of surgery including the immediate postoperative in-hospital period. Our inclusion criteria were designed so that study participants with at least 10 days of lead-in time could then complete at least 5 h of training before surgery, with the understanding that there may be unequal durations of gameplay based on scheduling patterns among patients. The training instrument used in this study, Lumosity, was created by Lumos Labs Inc., San Francisco, CA, USA, as a web-based and mobile platform designed to improve performance in specific cognitive abilities through interactive gameplay. As part of a customized training program tailored towards cognitive abilities thought to be deficient in the postoperative period, patients in the intervention arm will train in each of the following categories per session: memory, attention, problem solving, flexibility, and processing speed. Participants in the intervention arm will be given a study iPad that is WiFi enabled and locked to the Lumosity program. Adherence to the study protocol will be quantified using automated gameplay data received from Lumos Labs. Unblinded study members will be responsible for providing technical support to participants as needed and for periodic checks on adherence between in-person assessments.

### Control arm: usual care

Patients randomized to the control group will undergo the usual standard of care for cardiac surgical procedures, which currently does not consist of a perioperative neurocognitive training regimen. Instead of using an active control group as is common for the evaluation of the efficacy of a cognitive training instrument, the investigators thought that the assignment of a usual care control was more appropriate in this setting, as it is representative of a standard of care that currently lacks specific recommendations for cognitive activities in the perioperative period. Patients in the control arm will be asked to refrain from obtaining a Lumosity account to reduce any potential contamination with the intervention arm.

#### Study outcomes

##### Primary outcome: feasibility

To best evaluate the potential effect of a neurocognitive training program on the development of postoperative delirium and POCD, a large randomized controlled trial would need to be performed. However, before undertaking such a trial, it is imperative to show that implementation of a prehabilitative program is feasible. This is especially true given the heavy reliance on a patient-performed intervention and need for recruitment of interested subjects with sufficient lead-in time for the intervention to be effective. Furthermore, since this is the first time neurocognitive training will be implemented in this setting, there is no preliminary data regarding a potential effect size for an accurate power calculation to be performed. Therefore, the PEaPoD trial was designed to evaluate feasibility as a primary outcome. Feasibility will be assessed through both recruitment and adherence. Satisfactory recruitment will be defined as enrollment of 50% or more of eligible patients, which will reflect efficient screening and approach procedures and also sufficient interest within this patient population. Adherence will be quantified for analysis using automated gameplay data recorded during use. Consideration of three separate training periods (preoperative, immediate postoperative, and extended postoperative periods) will be taken into account in evaluating optimal adherence patterns for potential future study.

##### Secondary outcomes: postoperative delirium and POCD

Additional information will be obtained to inform future investigations. The incidence of postoperative delirium will be evaluated in both groups through the use of the CAM, the current standard for the diagnosis of delirium in clinical research studies [22]. Assessments will be conducted by study members blinded to group assignment on each postoperative day until the day of hospital discharge, or day seven, whichever comes first. In the event that a study patient is still intubated and/or is unable to speak, the CAM-ICU, a variation of the CAM that does not require verbal responses, will be used. Patients who refuse assessments will be approached again within a few hours, but they will not be coded as delirious for that day if the exam cannot be completed. Blinded team members have been extensively trained in the administration of the CAM by the group responsible for the creation of the long CAM assessment as part of a larger collaboration within the departments of Anesthesia and Medicine at BIDMC. Scoring of CAM assessments will be informed by the performance on basic cognitive tasks (immediate and delayed recall, orientation, abstraction, and attention), the Delirium Symptom Interview (DSI), and a daily pain assessment. Acute changes in performance in these cognitive areas will be compared to performance on baseline MoCA testing for reference. The occurrence of POCD will be evaluated using the MoCA, a diagnostic instrument designed to detect mild cogntiive impairment and dementia with a high degree of sensitivity and specificity [23]. The full MoCA has three distinct versions, each of which will be used in the course of the study to minimize the impact of learning on test performance. A baseline MoCA will be performed on the day of enrollment, followed by an additional assessment using a different version that will take place before surgery on the day of the patient’s operation. The additional preoperative assessment will allow for identification of any changes in performance that could be potentially attributable to neurocognitive training as well as identification of an updated cognitive baseline. A third MoCA will be administered on the day of hospital discharge, again using a new test version. Additionally, a t-MoCA will be administered at 1, 3, and 6 months postoperatively to track any long-term postoperative cognitive deficits or time of return to baseline cognitive performance. The t-MoCA contains all of the components of the full MoCA with the exception of the questions requiring visual cues or drawing, and scores between the two tests can be normalized for comparison [24]. In lieu of using the t-MoCA throughout the study for consistency, we believe that the use of the full MoCA where possible may be more sensitive to mild cognitive impairment, as it contains more testing categories and could potentially identify issues with impaired visuospatial tasks. Our protocol will allow for assessment windows of 7 days for the 1 month follow-up and 14 days for the 3 and 6 months follow-up t-MoCAs. As for the CAM, all cognitive assessments will be administered and scored by trained study team members blinded to the subject’s group assignment.

##### Patient satisfaction survey

Patient satisfaction will be assessed via an electronic survey that will evaluate the subject’s satisfaction with their participation in the study and identify particular areas for improvement with regard to the study protocol. Upon completion of the study, patients will be asked to provide both structured and open-ended responses to describe their experience. This data will be analyzed both quantitatively and qualitatively to guide future research.

##### Data collection

In addition to the data collected involving our primary and secondary outcomes, we will also collect information regarding patient demographics, clinical variables such as age and gender, surgical and anesthetic details, intensive care unit (ICU) and hospital length of stay, and patient mortality at 30 days and 6 months. Study data will be collected and managed using REDCap electronic data capture tools hosted at BIDMC. REDCap is a secure, web-based application designed to support data capture for research studies [25]. Designated members of the research team will be responsible for building and maintaining the electronic case report form, as well as monitoring data entry for completeness, timeliness, and accuracy.

##### Reporting of compliance and adverse events

A specialist within the research group will monitor protocol compliance, occurrence, and reporting of adverse events to the IRB.

#### Statistical analysis

##### Sample size calculation

A convenience sample of 45 patients will be chosen to assess the feasibility of perioperative neurocognitive training. A total of 20 enrolled and randomized subjects are needed per group to assess the rates of participation, adherence, and dropout associated with the study. To account for patients who may fail the initial MOCA screen or withdraw, we have added five patients to the desired enrollment sample size for a total planned enrollment of 45 patients.

##### Data analysis

Patients will be prospectively randomized using 1:1 block randomization. Analyses will be conducted using SAS version 9.4 (SAS Institute Inc., Cary, NC, USA) or later. Descriptive statistics of the data will be performed. Continuous data will be represented using mean (± standard deviation) or median (interquartile range) for variables not normally distributed and compared using parametric or non-parametric tests as appropriate. Categorical data will be presented as frequencies and proportions and compared using a chi-square or Fisher’s Exact test.

##### Analysis of the primary outcome

The primary outcome of the study is feasibility. Feasibility will be defined in terms of recruitment and adherence to protocol. Descriptive statistics of the data will be presented, including number of eligible patients and overall study consent rates. Reasons for declining participation will be documented and reported in aggregate. Adherence to the protocol will be assessed using automated data reports of user activity generated in collaboration with Lumosity. Per protocol, patients are expected to complete the intervention twice daily for at least 10 days preoperatively and 4 weeks postoperatively unless they are intubated or otherwise incapacitated due to critical illness. Adherence will be reported as the proportion of minutes completed over the total minutes required per protocol (calculated as 30 × [number of preoperative days + 28] minutes). Data will be reported for both the entire perioperative period, as well as individually for the preoperative and postoperative periods.

##### Analysis of secondary outcomes

Differences in the incidence of postoperative delirium and POCD will be presented as proportions and assessed with the use of a chi-square test. Odds ratios and 95% confidence intervals will be generated using logistic regression and interpreted. A parametric *t* test or non-parametric equivalent will be utilized to identify differences in POCD (MoCA scores) at each time point, controlling for baseline cognitive function. To account for the multiple measurements per subject, we will employ repeated measures regression techniques (e.g., analysis of variance). Although randomization should eliminate baseline confounders, univariate and multivariable logistic and linear regression modeling may be employed to assess the relationship between preoperative Lumosity use and both delirium and POCD, adjusting for any differences that may persist between groups.

## Discussion

The potential for neurocognitive training to prevent or mitigate the effects of cognitive deficits associated with various acute and chronic disease states has been investigated in other patient groups, but PEaPoD is the first study to do so in cardiac surgical patients, a subset of the population at highest risk for the development of postoperative delirium and POCD. Additionally, this novel study focuses on a prehabilitative intervention, whereas the majority of the work in the field focuses on alternative anesthetic and analgesic strategies, postoperative behavioral interventions, or cognitive rehabilitation after the insult has already occurred. Should the concept of perioperative neurocognitive training prove to be feasible through the conduct of this study, it will provide the framework for rigorous and efficient investigation into the technique’s efficacy as a low-risk, patient-led intervention for the prevention of significant perioperative morbidity affecting hundreds of thousands of patients in the USA alone on an annual basis.

Our study’s main limitation is that is not powered to detect a difference in clinical outcomes. Before a rigorous investigation into the efficacy of neurocognitive training in reducing postoperative delirium and POCD can be performed, the feasibility of our intervention in this population needs to be determined. While our sample size is larger than some would consider necessary for evaluating feasibility, it will provide accurate information as to an estimated timeline for a larger study and guide decision making as to whether a multicenter efficacy trial is a logical next step, as well as provide an estimate of effect size which can be used to power larger studies. Another limitation comes in the selection of the MoCA as our instrument for measuring POCD, a condition for which it has yet to be extensively validated. POCD is usually evaluated using an in-depth neuropsychological test battery. The MoCA provides multiple potential advantages over this approach in this setting. First, it is shorter, taking 15–20 min rather than 45–60 min to complete. Second, it is highly sensitive in detecting mild cognitive deficits. Third, it has multiple versions including a telephonic equivalent, minimizing the learning effect on test performance and providing a means for longitudinal comparison over a 6-month study period without the need for additional in-person patient visits. Most importantly, it assesses many of the cognitive areas of interest in the evaluation of the patient with potential POCD including attention, memory, and language.

In addition to these limitations, a potential source of bias in our study is the possibility for our assessors to become unblinded to group assignment throughout the course of a 6-month study period. We have taken many steps to prevent this occurrence and have systems in place to manage unblinding if it does occur. Throughout the course of the study, unblinded investigators will reiterate the importance of maintaining the blind, including detailed conversations both at the time of consent and prior to blinded study interactions (e.g., in-hospital cognitive assessments) on how to avoid potential conversations regarding the use of a mobile device in general. In an effort to maintain consistency, clearly defined roles have been delineated within the research group, with two unblinded team members solely responsible for monitoring adherence and technical support who are never involved in the assessment process. If an assessor does become unblinded, an alternate blinded team member trained in administering the CAM and MoCA is made available. If an alternate blinded team member is not available, the assessment is not performed and will be treated as missing data.

To summarize, elderly patients undergoing cardiac surgery have the highest incidence of postoperative delirium and POCD, which places them at an elevated risk for significantly higher perioperative morbidity and long-lasting detrimental effects on cognition. Neurocognitive training has been shown in preliminary studies to be effective in improving cognitive performance in the areas thought to be affected in the postoperative period. PEaPoD will be the first study to investigate the use of perioperative neurocognitive training in this high-risk patient population. Through the performance of this study, valuable information will be obtained that will guide future studies that are well designed to examine the technique’s efficacy in preventing postoperative delirium and POCD.

## Additional file

Additional file 1: SPIRIT checklist. (DOC 121 kb)

## Abbreviations

ACTIVE: Advanced Cognitive Training for Independent and Vital Elderly
BIDMC: Beth Israel Deaconess Medical Center
CAM: Confusion Assessment Method
CARE: Center for Anesthesia Research Excellence
CONSORT: Consolidated Standards of Reporting Trials
CPB: Cardiopulmonary bypass
HELP: Hospital Elder Life Program
ICU: Intensive care unit
IRB: Institutional Review Board
MoCA: Montreal Cognitive Assessment
PEaPoD: Prevention of Early Postoperative Decline
POCD: Postoperative cognitive decline
REDCap: Research Electronic Data Capture
SPIRIT: Standard Protocol Items: Recommendations for Interventional Trials
t-MoCA: Telephonic Montreal Cognitive Assessment

## References

[1] Inouye SK (2016). The short-term and long-term relationship between delirium and cognitive trajectory in older surgical patients. Alzheimers Dement.

[2] Rudolph JL, Marcantonio ER (2011). Review articles: postoperative delirium: acute change with long-term implications. Anesth Analg.

[3] Arrowsmith JE (2000). Central nervous system complications of cardiac surgery. Br J Anaesth.

[4] Newman MF (2001). Longitudinal assessment of neurocognitive function after coronary-artery bypass surgery. N Engl J Med.

[5] Etzioni DA (2003). The aging population and its impact on the surgery workforce. Ann Surg.

[6] Su X (2016). Dexmedetomidine for prevention of delirium in elderly patients after non-cardiac surgery: a randomised, double-blind, placebo-controlled trial. Lancet.

[7] Inouye SK, Westendorp RG, Saczynski JS (2014). Delirium in elderly people. Lancet.

[8] Mayo NE (2011). Impact of preoperative change in physical function on postoperative recovery: argument supporting prehabilitation for colorectal surgery. Surgery.

[9] Debes C, Aissou M, Beaussier M (2014). Prehabilitation. Preparing patients for surgery to improve functional recovery and reduce postoperative morbidity. Ann Fr Anesth Reanim.

[10] Kawano T (2015). Impact of preoperative environmental enrichment on prevention of development of cognitive impairment following abdominal surgery in a rat model. Anesthesiology.

[11] Tow A (2016). Cognitive reserve and postoperative delirium in older adults. J Am Geriatr Soc.

[12] Anguera JA (2013). Video game training enhances cognitive control in older adults. Nature.

[13] Bejjanki VR (2014). Action video game play facilitates the development of better perceptual templates. Proc Natl Acad Sci U S A.

[14] Edwards JD (2017). Speed of processing training results in lower risk of dementia. Alzheimers Dement.

[15] Ballesteros S (2014). Brain training with non-action video games enhances aspects of cognition in older adults: a randomized controlled trial. Front Aging Neurosci.

[16] Mayas J (2014). Plasticity of attentional functions in older adults after non-action video game training: a randomized controlled trial. PLOS One.

[17] Hardy JL (2015). Enhancing cognitive abilities with comprehensive training: a large, online, randomized, active-controlled Trial. PLOS One.

[18] Des Roches CA, Mitko A, Kiran S (2017). Relationship between self-administered cues and rehabilitation outcomes in individuals with aphasia: understanding individual responsiveness to a technology-based rehabilitation program. Front Hum Neurosci.

[19] Pressler SJ (2011). Nurse-enhanced memory intervention in heart failure: the MEMOIR study. J Card Fail.

[20] Chan AW (2013). SPIRIT 2013 statement: defining standard protocol items for clinical trials. Ann Intern Med.

[21] Altman DG (2001). The revised CONSORT statement for reporting randomized trials: explanation and elaboration. Ann Intern Med.

[22] Inouye SK (1990). Clarifying confusion: the confusion assessment method. A new method for detection of delirium. Ann Intern Med.

[23] Nasreddine ZS (2005). The Montreal Cognitive Assessment, MoCA: a brief screening tool for mild cognitive impairment. J Am Geriatr Soc.

[24] Pendlebury ST (2013). Telephone assessment of cognition after transient ischemic attack and stroke: modified telephone interview of cognitive status and telephone Montreal Cognitive Assessment versus face-to-face Montreal Cognitive Assessment and neuropsychological battery. Stroke.

[25] Harris PA (2009). Research electronic data capture (REDCap)—a metadata-driven methodology and workflow process for providing translational research informatics support. J Biomed Inform.

